# The Nucleic Acid of Partially Purified Rous No. 1 Sarcoma Virus

**DOI:** 10.1038/bjc.1957.75

**Published:** 1957-12

**Authors:** R. Bather


### THE NUCLEIC ACID OF PARTIALLY PURIFIED ROUS NO. 1 SARCOMA VIRUS

R. BATHER

*From the British Empire Cancer Campaign Unit, Poultry Research Centre, West Mains Road, Edinburgh, 9*

Received for publication August 20, 1957

ALTHOUGH tumour viruses have so far not been isolated in highly purified form, it seems likely that most of the material in the partially purified preparations obtained by repeated centrifugation and enzyme treatment (Carr and Harris, 1951 ; Bather, 1953) or centrifugation and protamine sulphate precipitation (Moloney, 1955) comes only from the cytoplasm of tumour cells. It is of interest therefore, to investigate the nucleic acid (N.A.) of such preparations using the newer methods of extraction and chromatography that have been applied with success to influenza virus (Ada and Perry, 1954). The present paper describes some preliminary observations on the chemical composition and nucleic acid content of Rous sarcoma virus concentrates and of cytoplasmic particle concentrates from normal fowl tissues.

### MATERIAL AND METHODS

Chickens from the highly susceptible inbred line of Brown Leghorns maintained at this Centre were used throughout. These birds are constantly selected for susceptibility to Rous sarcoma cells and virus, and allow infectivity titrations to be made in young chicks to a constant end-point. No negatives appear in any of the groups preceding that inoculated with the highest active dilution. The method of Parker and Rivers (1936) can be used to estimate the second figure in the end-point dilution. Groups of 4 or 5 chicks were used for each serial tenfold dilution of virus.

### *Preparation of partially purified virus and cytoplasmic particulate concentrates*

Most of the preparations were made by the standard method used in previous work (Bather, 1953) which was adapted from that originally devised by Carr and Harris (1951). The method employs fractional centrifugation and treatment with the enzymes trypsin and hyaluronidase. The relative centrifugal forces used throughout were 3500 g. for 20 minutes in a swinging cup rotor for clarification and 20,000 g. for 45 minutes in a Servall SS.1 angle head centrifuge for sedimentation of the virus and cytoplasmic particulates. On three occasions, protamine sulphate precipitation (Moloney, 1955) was used and gave final pellets which contained similar amounts of material to that obtained by the standard procedure (see " Experimental "). The concentrates were transferred to weighed test-tubes and stored in the deep-freeze until needed for further study. Infectivity titrations were always made on a sample of suspension immediately prior to the final sedimentation.

*Isolation and estimation of lipid and nucleic acid*

The virus or particulate concentrates were removed from the deep freeze and dried from the frozen state *in vacuo*. Lipids were extracted from the dried material with two washings of chloroform : ethanol mixture (2 : 1) followed by two washings with dry ether. The residue was dried *in vacuo* and weighed, and lipid content estimated by difference. The lipid extracts were pooled and in some cases the solvents were evaporated off and phosphorus determinations made on the residues. Five determinations made on Rous virus lipids gave an average of 2·2 per cent P with a range of 1·6–2·4 per cent. The average figure agrees closely with that found by Claude (1939) and indicates that approximately half of the lipid material is in the form of phospholipids. [Recent work of Moloney (1957) has pointed up the importance of oxidized phospholipids in the inactivation of Rous sarcoma virus.]

The lipid-free residues were extracted with 10 per cent sodium chloride at 100° C. for thirty minutes as described by Ada and Perry (1954). The residue was centrifuged after extraction and washed twice with 10 per cent sodium chloride. The extracts were pooled and diluted appropriately for reading in the Ultra Violet Spectrophotometer (Unicam SP.500). Readings were made over the wavelength range of 240–300 m$\mu$ and a standard curve was prepared using a purified sample (Smith and Markham, 1950) of commercial yeast nucleic acid. The purified yeast N.A. contained 8·16 per cent phosphorus. Blank readings were made on a 10 per cent sodium chloride extract of normal chicken plasma protein. The fit of the corrected U.V. absorption curves of virus and particulate N.A. were very close to that of the purified material (Fig. 1).

The salt extracted residues from three different virus preparations were further extracted with 1 N perchloric acid at 80° C. for thirty minutes. No evidence of residual N.A. was found in the perchloric acid extracts when examined in the U.V. Spectrophotometer (Fig. 1).

Estimates of the amounts of N.A. in the NaCl extracts were made by applying the factor 9·7 calculated from the known proportions of bases (see " Experimental ") to convert their phosphorus content to N.A. content.

*Determination of the molecular proportions of nitrogenous bases in the nucleic acids*

The N.A. extracted as described was precipitated from the 10 per cent NaCl solution by the addition of two volumes of 96 per cent ethyl alcohol and a few drops of 0·1 N HCl to reduce the pH to approximately 2. Precipitation was allowed to take place at 0–4° C. for at least 24 hours. The precipitates were washed in 66 per cent, then 96 per cent ethanol and, finally, dry ether, and dried. 1·0 N HCl was added so that the final concentration of N.A. was about 10 mg./ml. (usually 40–50 $\mu$l. were required) and the tubes sealed. Hydrolysis was carried out at 100° C. for one hour. The hydrolysates were spotted on paper strips washed according to the method of Hanes, Hird and Isherwood (1952). The strips were usually 1·0–1·5 cm. wide. The hydrolyzed bases were separated by ascending chromatography in the solvent mixture isopropanol—HCl—water (65–18–17) (Wyatt, 1951).

The bases were located by U.V. light using a Chromatolite Fluorescence Lamp (Scottish Instruments) and cut out, together with corresponding pieces from a blank strip and eluted in 5 ml. 0·1 N HCl for 24 hours. A sample of hydrolyzed yeast N.A. was always run with each series of virus or particulate N.A.'s. The concentration of each purine or pyrimidine nucleotide was estimated by its absorption in the U.V. Spectrophotometer. Absorption at the maxima were

FIG. 1.—Ultra violet absorption curves of purified yeast R.N.A., partially purified Rous virus R.N.A. and perchloric acid extract of salt extracted Rous virus preparation.

●———● Partially purified Rous virus R.N.A.
○- - - -○ Purified yeast R.N.A.
×- - - - -× Perchloride acid extract of salt extracted Rous virus preparations.

determined for $10^{-3}$ M solutions or each of the purine bases and pyrimidine nucleotides using pure preparations (Light & Co.).

The values obtained were :

| | | | |
|---|---|---|---|
| Guanine | $E_{250}$ | = | 11·0 |
| Adenine | $E_{260}$ | = | 13·0 |
| Cytidylic acid | $E_{280}$ | = | 12·5 |
| Uridylic acid | $E_{260}$ | = | 10·0 |

Other procedures will be discussed in the experimental section concerned.

### EXPERIMENTAL

### *Examination of the partially purified virus preparations in the electron microscope and the analytical ultra centrifuge*

Samples of the partially purified virus preparations were frequently taken after the final sedimentation. These were resuspended in water or phosphatecitrate buffer at pH 6·7 and sprayed on to grids for examination in a Phillips (25Å Model) Electron Microscope or a Spinco electrically driven analytical ultracentrifuge.

The electron micrographs usually showed particles of a fairly uniform size distribution. In some preparations large, diffuse spots were present which tended to fade at high voltage and may have been free lipoid material. No attempt at accurate measurement of the particles was made because of generally poor contrast and uncertainty as to the true nature of all the particles present. However, electron microscope examination of many of the concentrated preparations served to confirm a reasonable uniformity of particle size within the range expected for Rous sarcoma virus (50–100 m$\mu$) and it was not considered feasible at the present stage to expect more detailed information.

Rates of sedimentation of the partially purified virus preparations were determined using the Spinco ultra-centrifuge incorporating a modified Schlieren optical system. Experiments were carried out on highly active preparations of several concentrations between 0·05 and 0·3 per cent in 0·1 M phosphate : citrate buffer (pH 6·7). Speeds of 12,500 r.p.m. were used.

Within experimental error the sedimentation constant was independent of concentration and had the value of $470 \times 10^{-13}$ C.G.S. units. Using the newer value of Kahler, Bryan, Lloyd and Moloney (1954*a*) for the density of Rous sarcoma virus this leads to an average diameter for the particles of approximately 80 m$\mu$.

*Presence of normal cell components in partially purified Rous sarcoma virus preparations*

The normal cell contaminants most likely to be present after careful selection of viable tumour tissues are from blood, muscle fibres and, possibly, connective tissue cells. The author has observed that muscle cells yield only about 1/10 as much concentrated cytoplasmic material as tumour. Red blood cells contain little of this type of particle in their cytoplasm. White cells and lymphocytes may be expected to yield a considerable amount of cytoplasmic concentrate as is evident from the results for spleen (" Experimental " Section 4) but the numbers of these cells present must be small. There remains the possibility that, since Rous sarcoma is of connective tissue origin, there may remain in the tumour cells cytoplasmic material derived from normal connective tissue cells, which may contribute particles to the virus concentrates. Attempts were made to isolate a cytoplasmic particle concentrate from cock's comb, a tissue that is almost entirely composed of connective tissue cells. Microscopically, comb tissue presents a picture reminiscent of spindle-cell sarcoma with whorling bundles of connective tissue cells and, like the avian virus-induced tumours, yields considerable amounts of mucoid material which is degraded by hyaluronidase. As much as 48 g. of pooled comb tissue were homogenized and extracted in the same way as tumour tissue. The amount of concentrated material in the final pellet was minute and difficult to disperse. It was found to consist almost entirely of collagen fibres whose characteristic banded structure was clearly seen in the electron microscope.

It is clear, then, that any contribution by normal particles to the virus concentrates must be very small and even a figure of 1 per cent is probably too high.

*Identification of the purine-bound sugar in the nucleic acid of partially purified Rous virus preparations*

Three Rous virus concentrate preparations were analysed for the presence of deoxyribose by the method of Webb and Levy (1955) using *p*-nitrophenylhydrazine. No deoxyribose was detected despite the reported high sensitivity of this reagent.

The purine-bound sugar was released from partially purified Rous virus nucleic acid (prepared as described in "Methods") by digestion of approximately 500 μg. N.A. in 0·25 ml. normal $H_2SO_4$ in a sealed tube at 100° C. for one hour. The hydrolysate was neutralized with saturated $Ba(OH)_2$ and the supernatant evaporated to dryness. The resulting residue was extracted with hot 96 per cent ethanol and the extract evaporated to a small enough quantity for convenient spotting for paper chromatography. Two solvent systems were used, namely phenol and butanol-acetic acid. The isolated sugar was chromatographed with pure ribose, xylose and arabinose. In three experiments the purine-bound sugar from Rous virus concentrates corresponded in $R_F$ value with pure ribose. No deoxyribose or other purine sugar was evident.

*Lipid and ribonucleic acid content of partially purified Rous virus preparations and normal cytoplasmic particulate concentrates*

Lipids and nucleic acids were isolated and estimated as described in "Methods". The results are listed in Table I. The first set of values for Rous sarcoma are for concentrates prepared by the standard fractional centrifugation-enzyme digestion procedure described in the "Methods". The second set are for concentrates prepared as described by Moloney (1955) using protamine sulphate precipitation.

TABLE I.—*Lipid and Ribonucleic Acid Content of Partially Purified Rous Sarcoma Virus Preparations and Cytoplasmic Particulate Concentrates from Spleen, Liver and Brain*

| Tissue of origin | Observations | Virus and particulate yields mg./g. wet tissue | Per cent lipid ± S.D. | Per cent R.N.A. ± S.D. | Range of infectivity (M.I.D./g. tumour) |
|---|---|---|---|---|---|
| Rous sarcoma (1) | 38 | 0·51 | 42·5 ±9·0 | 1·42 ±0·37 | $10^{1.2}$–$10^{6.5}$ |
| Rous sarcoma (2) | 3 | 0·54 | 43·6* (37·2–50·0) | 0·78* (0·92–0·64) | $10^{2.5}$–$10^{4.5}$ |
| Spleen | 4 | 7·04 | 36·5 ±4·0 | 1·02 ±0·10 | — |
| Liver | 5 | 4·60 | 37·6 ±4·7 | 0·61 ±0·14 | — |
| Brain | 2 | 5·96 | 31·6 (32·7–30·4) | 0·44 (0·42–0·46) | — |

* Two determinations only.

Both sets of results are comparable, although the nucleic acid content is low in the protamine sulphate preparations. However, there is a wide variation in the R.N.A. content of tumour extracts prepared by either method (0·64–2·12 per cent) and this may be a reflection of the variation in infectivity of the different preparations. The range of infectivities available is not sufficient, as yet, to show whether or not a correlation exists with R.N.A. content of the type found for influenza virus (Ada and Perry, 1956). Liver and brain concentrates appear to have a lower average content of R.N.A. compared with Rous virus concentrates, brain showing the lowest. This could be due to the lesser activity of these tissues metabolically (especially in the case of brain) assuming that R.N.A. is intimately concerned in growth and cell division.

*Molecular proportions of nitrogenous bases in ribonucleic acid from various sources*

Ribonucleic acid isolated from partially purified virus preparations and normal cell particulate concentrates was hydrolyzed and chromatographed as described in the " Methods ". The molecular proportions, referred to Adenine as 1·00, were calculated from the U.V. absorption data of the eluted paper strips (Table II).

TABLE II.—*Molecular Proportions of Purine and Pyrimidine Bases obtained by Chromatographic Separation on Filter Paper of R.N.A. from Yeast, Partially Purified Rous sarcoma Virus Preparations and Cytoplasmic Concentrates of Fowl Liver and Spleen*

| Source of R.N.A. | Number of obs. | Adenine | Guanine ± S.D. | Cytosine ± S.D. | Uracil ± S.D. | $\frac{A+U}{G+C}$ ± S.D. | $\frac{Pu.}{Py.}$ ± S.D. | $\frac{A+C}{G+U}$ ± S.D. |
|---|---|---|---|---|---|---|---|---|
| Yeast . | 7 | 1·00 | 1·15 | 0·82 | 0·88 | 0·96 | 1·27 | 0·89 |
| | | | ±0·04 | ±0·01 | ±0·02 | ±0·02 | ±0·03 | ±0·02 |
| Rous | 6 | 1·00 | 1·80 | 1·55 | 0·81 | 0·54 | 1·20 | 0·98 |
| sarcoma | | | ±0·08 | ±0·03 | ±0·07 | ±0·03 | ±0·04 | ±0·03 |
| Spleen . | 4 | 1·00 | 1·98 | 1·54 | 0·81 | 0·52 | 1·27 | 0·92 |
| | | | ±0·11 | ±0·13 | ±0·08 | ±0·01 | ±0·05 | ±0·06 |
| Liver . | 2 | 1·00 | 1·81 | 1·58 | 0·71 | 0·50 | 1·22 | 1·02 |
| | | | (1·78–1·84) | (1·62–1·54) | (0·67–0·75) | (0·49–0·52) | (1·22–1·24) | (1·07–0·98) |

R.N.A. from Rous sarcoma concentrated extracts in common with that from spleen and liver, contains a large proportion of both guanine and cytosine. Such a preponderance of these two bases has been found in most ribonucleic acids studied with the exception of yeast and influenza virus R.N.A. (Elson and Chargaff, 1954 ; Ada and Perry, 1954, 1956). No significant differences have appeared in the ratios of $\frac{\text{adenine} + \text{uracil}}{\text{guanine} + \text{cytosine}}$ between Rous virus concentrate and spleen and liver R.N.A. when subjected to the $t$-test at the 1 per cent level. The final column $\left(\frac{\text{adenine} + \text{cytosine}}{\text{guanine} + \text{uracil}}\right)$ indicates that in all cases the number of 6-keto groups is nearly equal to the number of 6-amino groups and is of interest in relation to the possible configuration of the R.N.A. molecule (Elson and Chargaff, 1954).

### DISCUSSION

Previous estimates of the nucleic acid content of partially purified Rous virus concentrates were made by Claude (1939), who gave a value of 5–8 per cent of the total virus material as N.A., and by Shemin and Sproule (1942) who estimated the N.A. as approximately 1·5 per cent. The former based his values on U.V. absorption methods while the latter used purine nitrogen values. The experiments described here show that Rous virus concentrate R.N.A. can be extracted completely and in a relatively pure form with 10 per cent NaCl and that the amounts so obtained vary considerably between a minimum of 0·64 per cent and a maximum of 2·12 per cent of the total virus concentrate. Claude did not use trypsin digestion to further purify his material and it may be that the high value found was because of this.

Recent reports from Kahler *et al.* (1954*a*, *b*) have placed the density of Rous virus at approximately 1·15 or lower and the diameter at 90 m$\mu$. The data presented here indicate that the great majority of the particles in the partially purified virus preparations, with which almost all the infectivity is associated, are of fairly uniform size and density, as far as can be ascertained with the electron microscope and the analytical ultracentrifuge. Their diameter in these experiments was calculated as approximately 80 m$\mu$. The molecular weight of the Rous virus particle has been estimated to be approximately $140 \times 10^6$ (Claude, 1937). The values for R.N.A. content given in this paper show that Rous sarcoma virus fits in with the relationship pointed out by Frisch-Niggemeyer (1956) and Cheng (1957), namely that most R.N.A. containing viruses and cytoplasmic particles appear to contain the same absolute amount of R.N.A. (approximately $2 \times 10^6$ g. per mole of virus or particle).

No differences in the molecular proportions of purine and pyrimidine bases occurred between partially purified preparations of Rous virus and the cytoplasmic particulate concentrates of the normal tissues studied. The question of purity is, of course, ever present in tumour virus work and it may be argued that most of the nucleic acid came from non-tumour sources. It can be said, however, that normal cell components must be present in very small amounts in virus concentrates prepared in this way. The fact that no D.N.A. can be detected in the preparations makes improbable contamination by nuclear material. Non-virus protein contaminants are removed to a large extent by trypsin treatment. We are left, then, with a suspension of cytoplasmic particles derived almost entirely from tumour cells, a counterpart of which (cock's comb connective tissue) yields negligible quantities of such particles. The fact that the amounts of material in the high speed centrifuge pellets are different when isolated from different tissues probably reflects the variations in the fine structure of the cytoplasm. Palade (1955) and Palade an Siekevitz (1956) have studied many types of cell in the electron microscope and attempted to relate the fine structure to cytological, histochemical and cytochemical information. It is not known how much of the pellet material obtained in these experiments can be attributed to the very small (100–150Å) particulates described by Palade. If, as has been suggested, these particulates correspond to those isolated by Petermann and co-workers by differential centrifugation (1952, 1953 and 1954) then most of them would be discarded during the isolation of the partially purified virus and cytoplasmic particulates by the method employed here. In any case, as Petermann and co-workers have shown that in mouse leukaemia all their spleen cytoplasmic particulate fractions increased over the normal picture it is probable that particles isolated along with the virus are closely related to virus in the metabolic processes of the cell. The situation is further complicated by the fact that only a very few particles structurally resembling virus are seen in sectioned pellets of centrifuged extracts of Rous sarcoma (Bernhard, Oberling and Vigier, 1956). No guarantee, therefore, that all the nucleic acid isolated from the partially purified virus preparations is from virus can be given. It can only be said that it is intimately associated with the cytoplasmic particulates of Rous cells and with that fraction of the particulates which contains the infective principle.

It would be interesting to see if other virus-induced tumours yield concentrates containing different R.N.A. from that found in Rous, and several tumours which are now available at this Centre are being studied.

The lack of any marked difference between normal and virus nucleic acid may not be surprising when it is remembered that tumour viruses live in a peculiarly close symbiosis with the parent cell, usually remaining within the cell throughout the life of the tumour. Any differences there are in nucleic acid composition, therefore, may be so subtle as to be impossible to detect by methods such as were employed here.

### SUMMARY

The nucleic acid associated with partially purified preparations of Rous sarcoma virus can be quantitatively extracted and estimated using 10 per cent sodium chloride solution and ultra-violet spectrophotometry. The preparations used consist of particles of reasonably uniform size exhibiting a sharp peak in the analytical ultracentrifuge and having a sedimentation constant of 470 C.G.S. units and a calculated diameter of approximately 80 m$\mu$. They are almost entirely free of normal tissue and nuclear contaminants.

The purine-bound sugar contained in the nucleic acid obtained from the Rous sarcoma virus preparations has been identified by paper chromatography as ribose and no evidence of deoxyribose was found by chromatography or the *p*-nitrophenylhydrazine reaction.

A series of 38 Rous sarcoma virus preparations was found to contain 1·42 $\pm$ 0·37 per cent R.N.A. with a range of values extending from 0·64–2·12 per cent. The average R.N.A. content was higher than that of cytoplasmic particulate concentrates from spleen (1·02 $\pm$ 0·10 per cent), liver (0·61 $\pm$ 0·14 per cent) and brain (0·42–0·46 per cent).

No significant differences were found in the molecular proportions of purine and pyrimidine bases from Rous sarcoma virus preparations or cytoplasmic particulate concentrates from normal tissues.

All expenses in connection with this work were borne by the British Empire Cancer Campaign. I should like to thank Dr. C. T. Greenwood of the Department of Chemistry, King's Buildings, Edinburgh, for his co-operation in obtaining the results with the analytical ultracentrifuge.

### REFERENCES

ADA, G. L. AND PERRY, B. T.—(1954) *Aust. J. exp. Biol. med.*, **32**, 453.

*Iidem.*—(1956) *J. gen. Microbiol.*, **14**, 623.

BATHER, R.—(1953) *Brit. J. Cancer*, **7**, 492.

BERNHARD, W., OBERLING, CH. AND VIGIER, PH.—(1956) *Bull. Ass. franç. Cancer*, **43**, 407.

CARR, J. G. AND HARRIS, R. J. C.—(1951) *Ibid.*, **5**, 83.

CHENG, PING-YAO.—(1957) *Nature*, **179**, 426.

CLAUDE, A.—(1937) *J. exp. Med.*, **66**, 59.

*Idem.*—(1939) *Science*, **90**, 213.

ELSON, D. AND CHARGAFF, E.—(1954) *Nature*, **173**, 1038.
FRISCH-NIGGEMEYER, W.—(1956) *Ibid.*, **178**, 307.
HANES, C. S., HIRD, F. J. R. AND ISHERWOOD, F. A.—(1952) *Biochem. J.*, **51**, 25.
KAHLER, H., BRYAN, W. R., LLOYD, B. J. AND MOLONEY, J. B.—(1954*a*) *J. nat. Cancer Inst.*, **15**, 331.
*Iidem.*—(1954*b*) *Ibid.*, **15**, 337.
MOLONEY, J. B.—(1955) *Ibid.*, **16**, 877.
*Idem.*—(1957) *Ibid.*, **18**, 515.
PALADE, G. E.—(1955) *J. Biophys. Biochem. Cytol.*, **1**, 59.
*Idem* AND SIEKEVITZ, P.—(1956) *Ibid.*, **2**, 171.
PARKER, R. F. AND RIVERS, T. M.—(1936) *J. exp. Med.*, **64**, 439.
PETERMANN, M. L. AND HAMILTON, M. G.—(1952) *Cancer Res.*, **12**, 373.
*Idem*, MIZEN, N. A. AND HAMILTON, M. G.—(1953) *Ibid.*, **13**, 372.
*Idem*, HAMILTON, M. G. AND MIZEN, N. A.—(1954) *Ibid.*, **14**, 360.
SHEMIN, D. AND SPROULE, E. E.—(1942) *Ibid.*, **2**, 514.
SMITH, J. D. AND MARKHAM, R.—(1950) *Biochem. J.*, **46**, 509.
WEBB, J. M. AND LEVY, H. B.—(1955) *J. biol. Chem.*, **213**, 107.
WYATT, G. R.—(1951) *Biochem. J.*, **48**, 584.


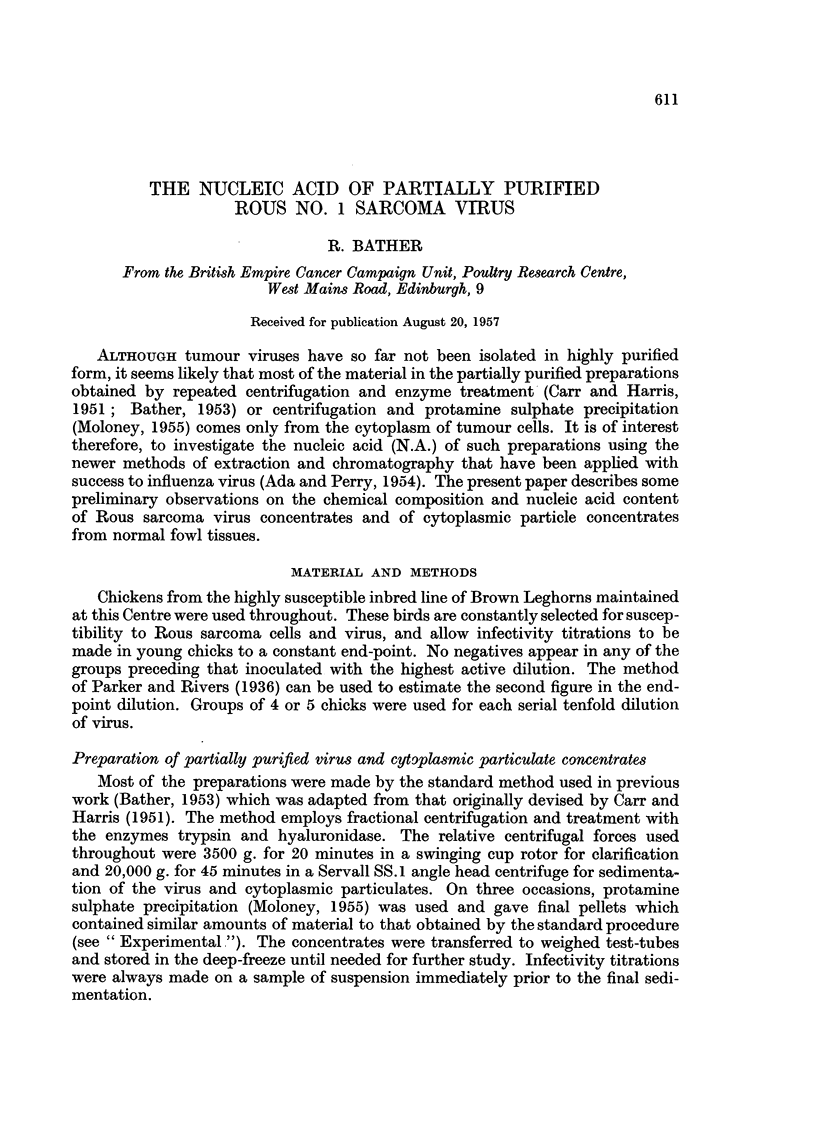

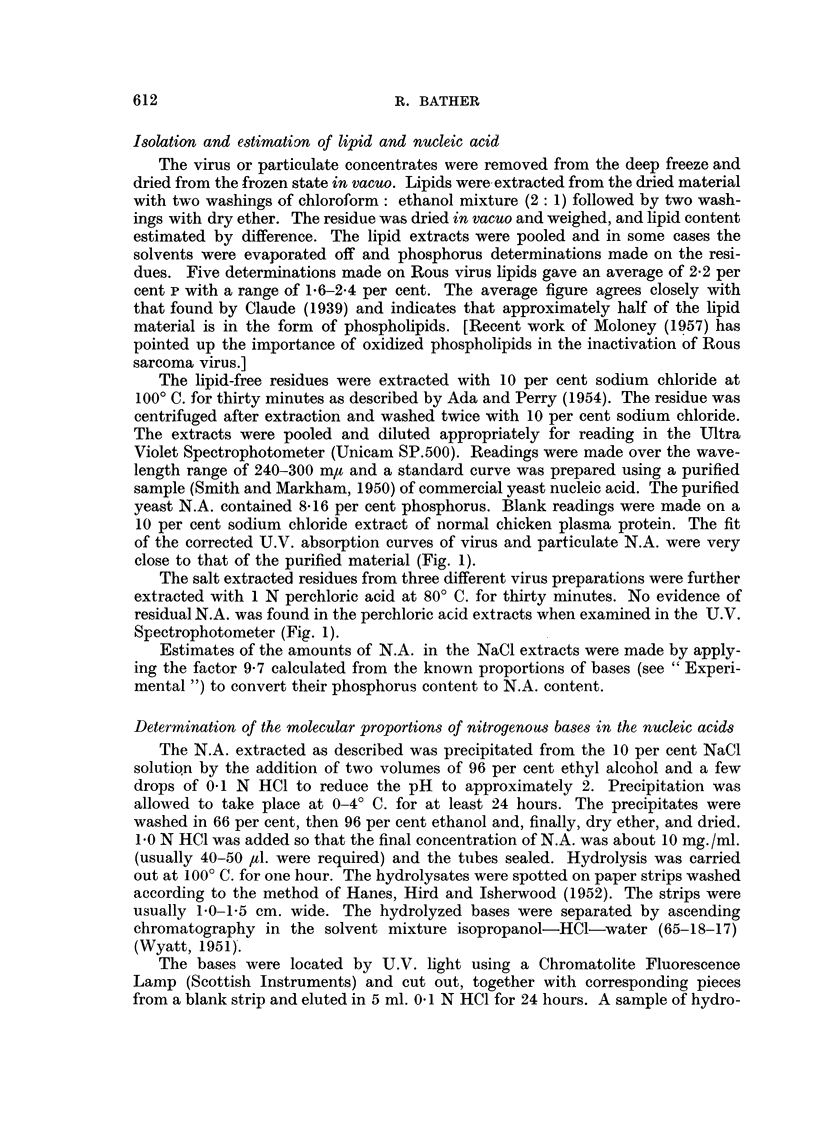

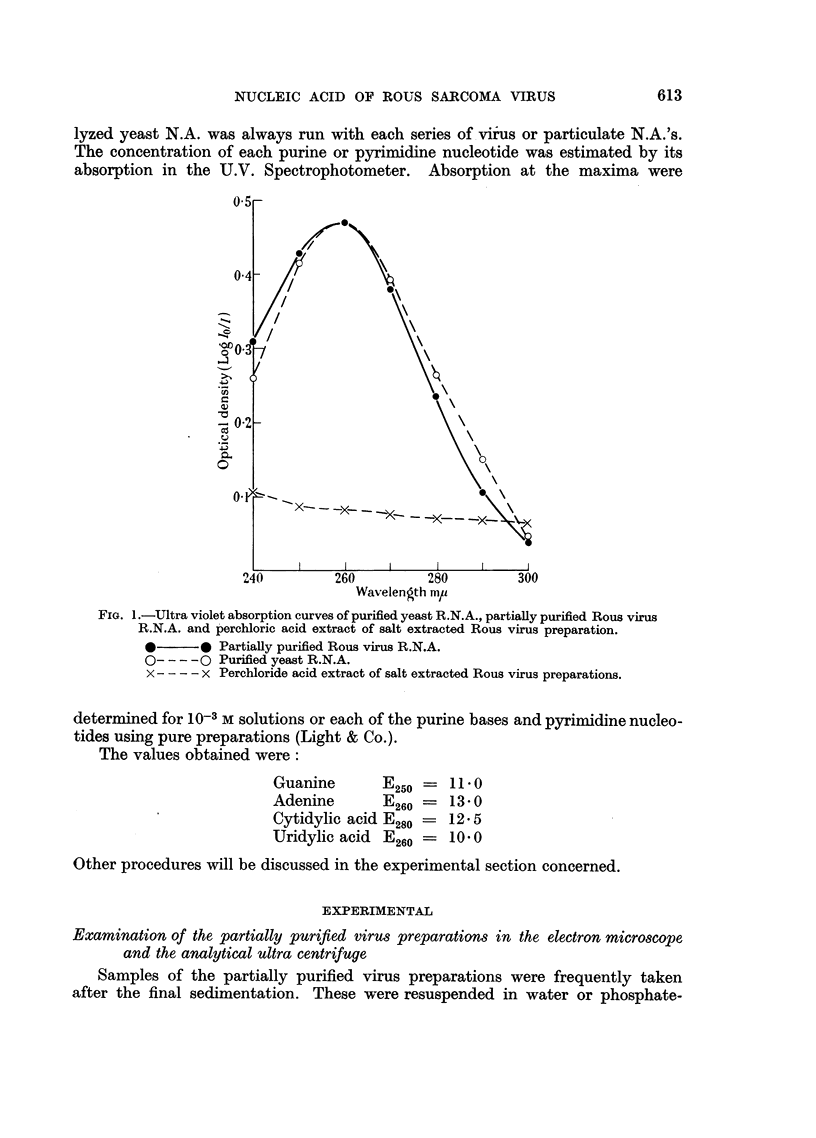

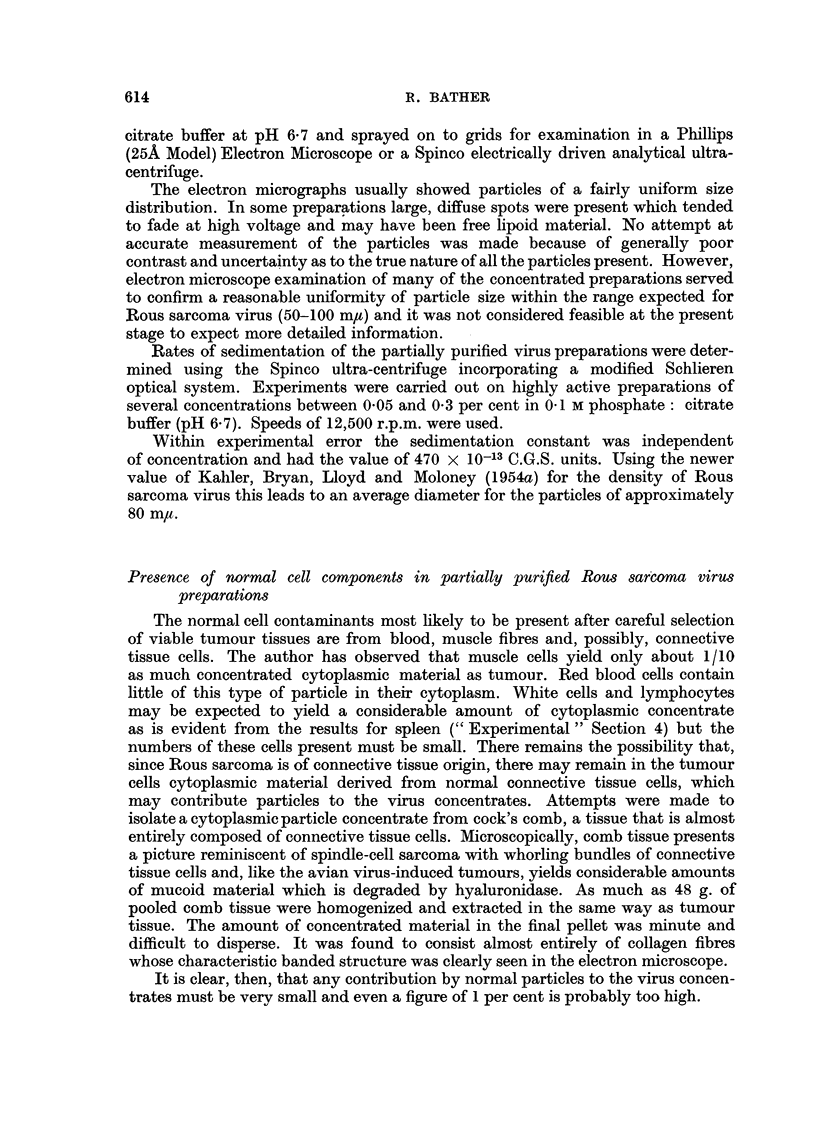

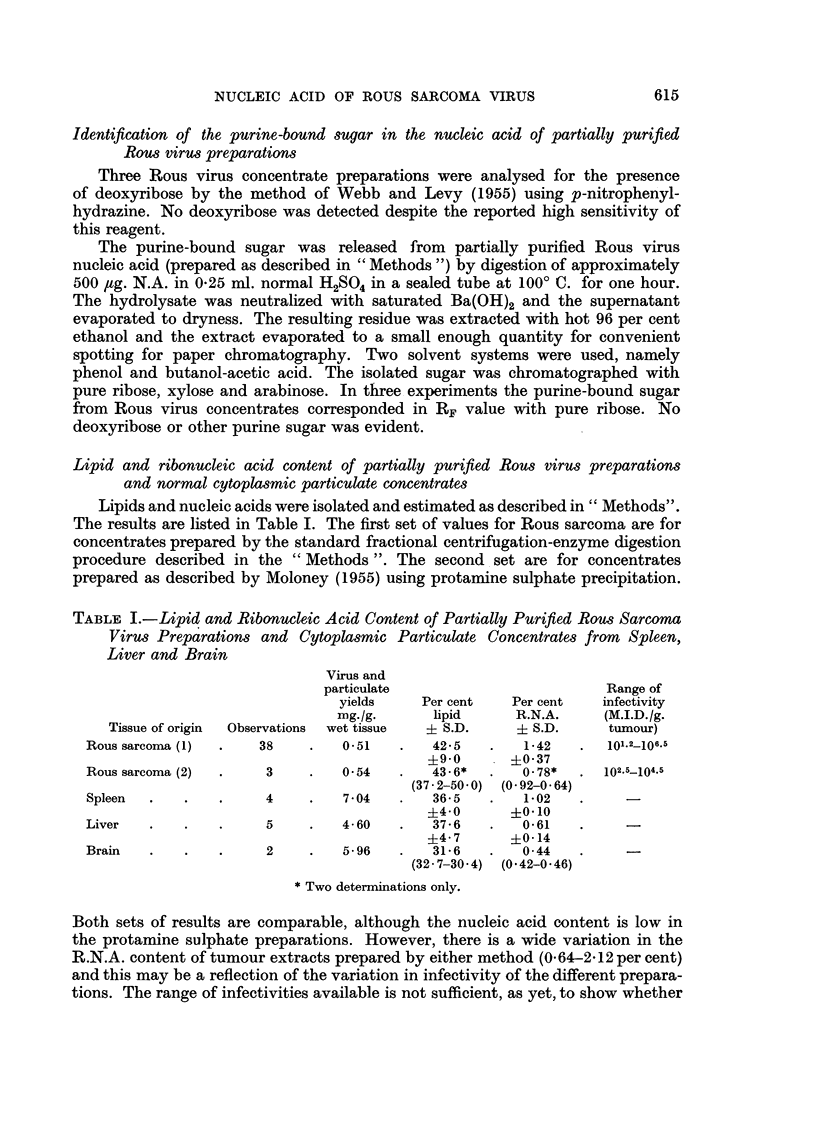

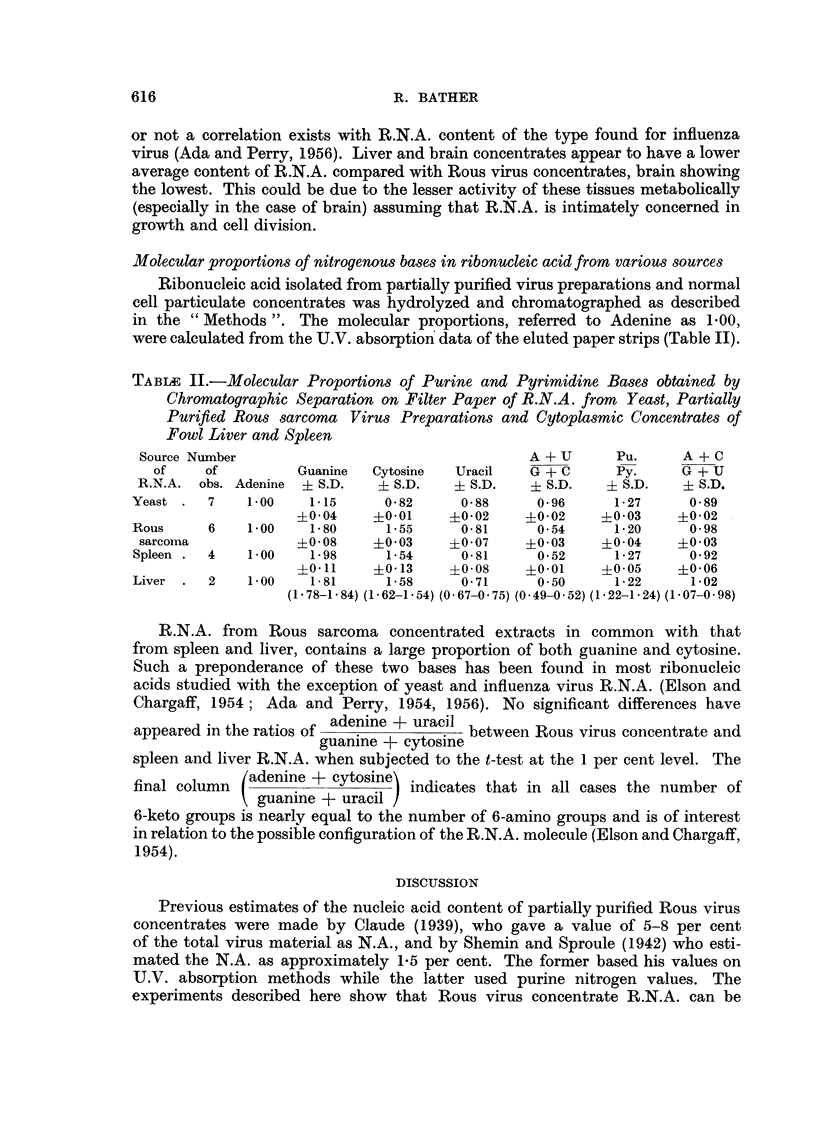

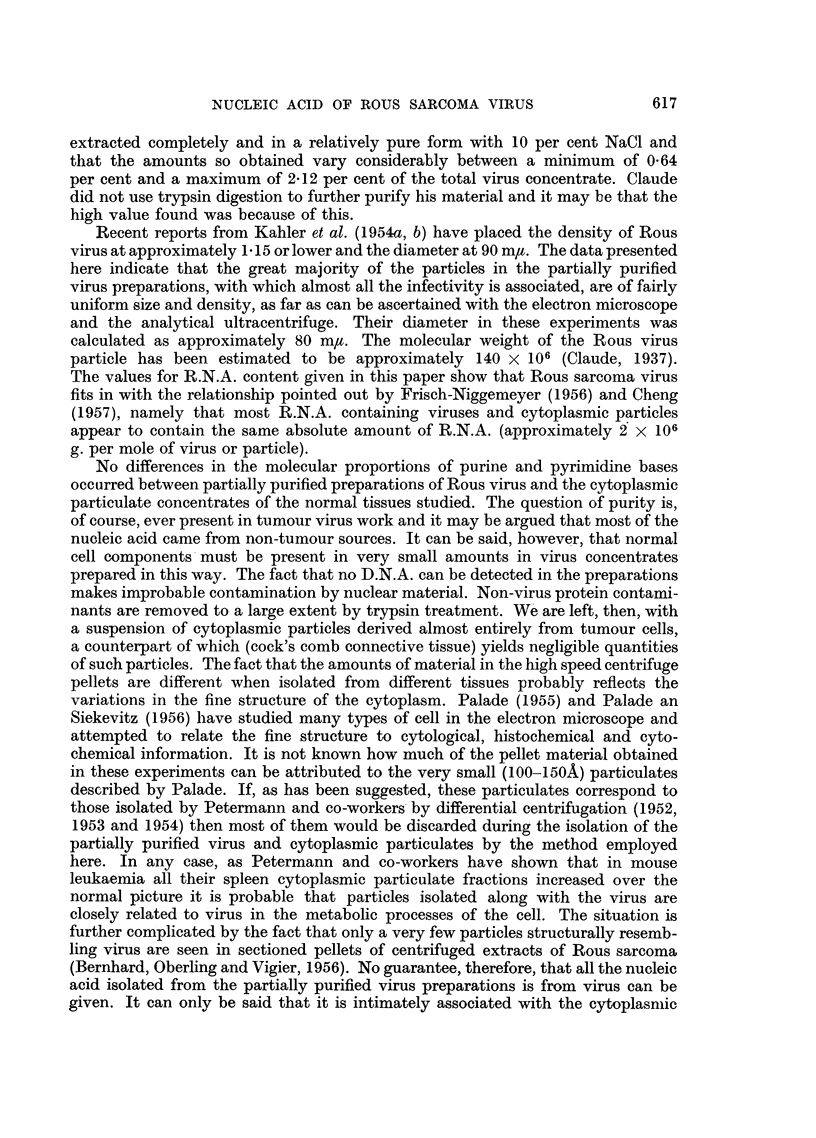

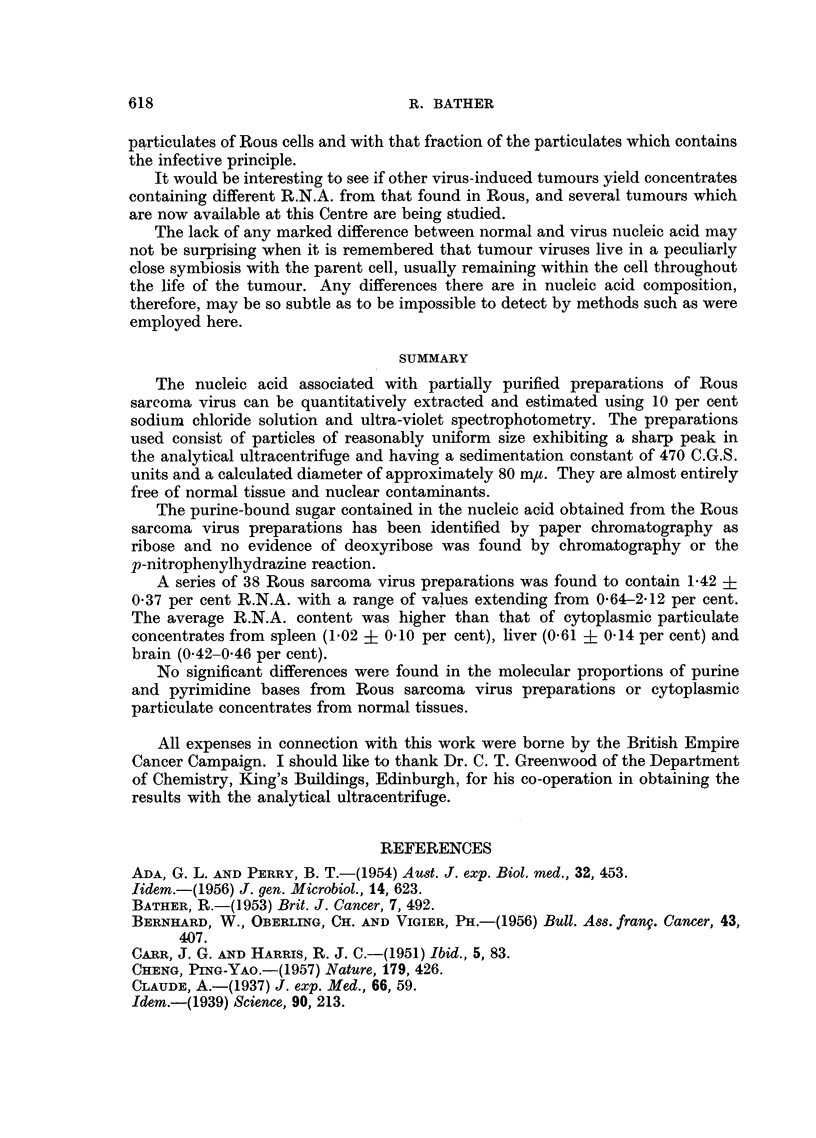

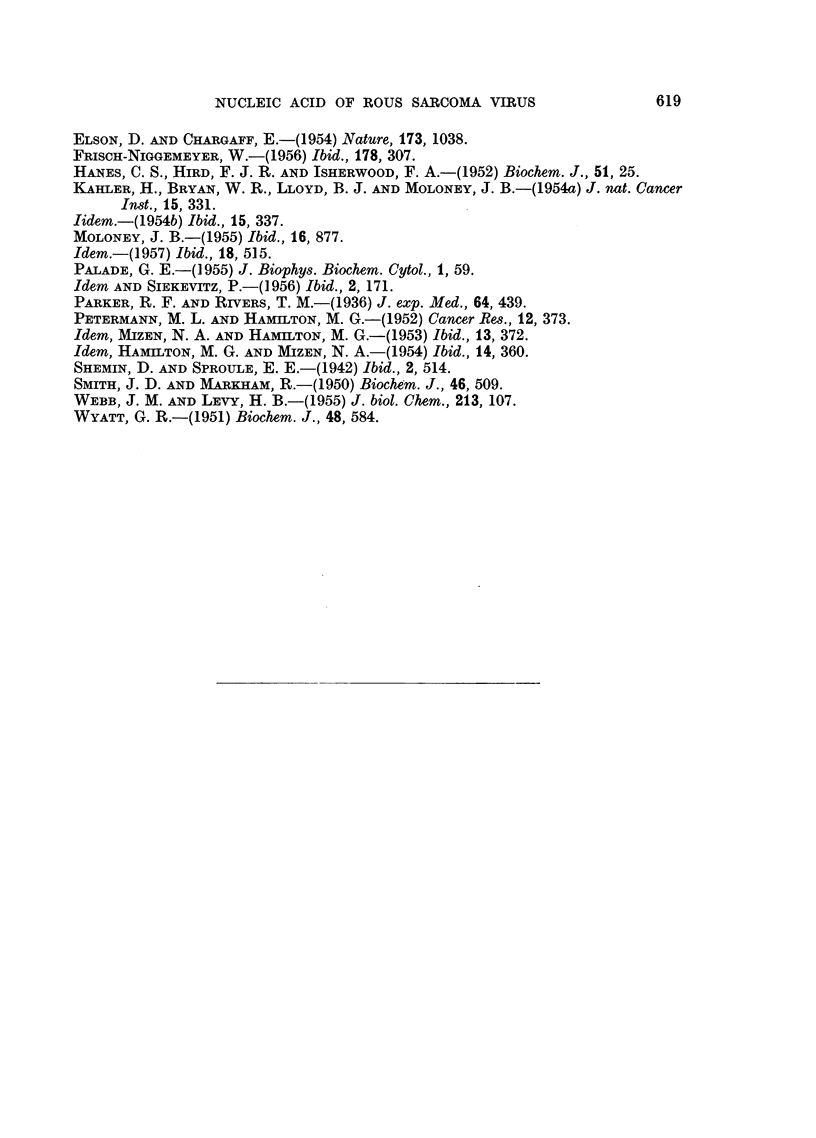


## References

[OCR_00458] ADA G. L., PERRY B. T. (1956). Influenza virus nucleic acid: relationship between biological characteristics of the virus particle and properties of the nucleic acid.. J Gen Microbiol.

[OCR_00461] BATHER R. (1953). Relation between the amount of virus material and infectivity of Rous No. 1 sarcomata.. Br J Cancer.

[OCR_00469] CHENG P. Y. (1957). Absolute amount of ribonucleic acid in virus and cytoplasmic particles.. Nature.

[OCR_00475] FRISCH-NIGGEMEYER W. (1956). Absolute amount of ribonucleic acid in viruses.. Nature.

[OCR_00481] KAHLER H., BRYAN W. R., LLOYD B. J., MOLONEY J. B. (1954). The density of the Rous sarcoma virus in sucrose solutions.. J Natl Cancer Inst.

[OCR_00488] PALADE G. E., SIEKEVITZ P. (1956). Liver microsomes; an integrated morphological and biochemical study.. J Biophys Biochem Cytol.

[OCR_00495] PETERMANN M. L., HAMILTON M. G., MIZEN N. A. (1954). Electrophoretic analysis of the macromolecular nucleoprotein particles of mammalian cytoplasm.. Cancer Res.

[OCR_00493] PETERMANN M. L., MIZEN N. A., HAMILTON M. G. (1953). The macromolecular particles of normal and regenerating rat liver.. Cancer Res.

[OCR_00498] SMITH J. D., MARKHAM R. (1950). Chromatographic studies on nucleic acids; the quantitative analysis of ribonucleic acids.. Biochem J.

[OCR_00500] WEBB J. M., LEVY H. B. (1955). A sensitive method for the determination of deoxyribonucleic acid in tissues and microorganisms.. J Biol Chem.

[OCR_00501] WYATT G. R. (1951). The purine and pyrimidine composition of deoxypentose nucleic acids.. Biochem J.

